# Supplementary material for: Complexome profiling of the Chlamydomonas psb28 mutant reveals TEF5 as an early PSII assembly factor
Source: Plant Cell. 2025 Mar 17;37(6):koaf055. doi: 10.1093/plcell/koaf055 (PMC12164590; doi:10.1093/plcell/koaf055)
Supplement: koaf055_Supplementary_Data [file koaf055_supplementary_data.zip › psb28_interactive.zip!/plots/intensity/qualityPlots/peptides/RPL26_Cre01.g040000.t1.2.html]

### Cre01.g040000.t1.2

Trivial name: RPL26  
SeqCoverage [median]: 21.40  
Mass: 16.47 kDa  
  
 

| Sequence | MissedCleavage | Mass [Da] | wtIntensity | muIntensity | Score |
| --- | --- | --- | --- | --- | --- |
| AVPIRKDDEVQVTR | 2 | 1624.89 | 1034.30 | 1730.40 | 70.62 |
| KAHFSAPSNVR | 1 | 1212.64 | 0.00 | 414.25 | 35.20 |
| VVEVYR | 0 | 763.42 | 0.00 | 795.22 | 80.38 |
|  |  |  | 1034.3 | 2939.9 |  |
